# MMP-Sensitive PEG Diacrylate Hydrogels with Spatial Variations in Matrix Properties Stimulate Directional Vascular Sprout Formation

**DOI:** 10.1371/journal.pone.0058897

**Published:** 2013-03-12

**Authors:** Michael V. Turturro, Megan C. Christenson, Jeffery C. Larson, Daniel A. Young, Eric M. Brey, Georgia Papavasiliou

**Affiliations:** 1 Department of Biomedical Engineering, Illinois Institute of Technology, Chicago, Illinois, United States of America; 2 Research Service, Edward Hines Jr. VA Hospital, Hines, Illinois, United States of America; University of California, San Diego, United States of America

## Abstract

The spatial presentation of immobilized extracellular matrix (ECM) cues and matrix mechanical properties play an important role in directed and guided cell behavior and neovascularization. The goal of this work was to explore whether gradients of elastic modulus, immobilized matrix metalloproteinase (MMP)-sensitivity, and YRGDS cell adhesion ligands are capable of directing 3D vascular sprout formation in tissue engineered scaffolds. PEGDA hydrogels were engineered with mechanical and biofunctional gradients using perfusion-based frontal photopolymerization (PBFP). Bulk photopolymerized hydrogels with uniform mechanical properties, degradation, and immobilized biofunctionality served as controls. Gradient hydrogels exhibited an 80.4% decrease in elastic modulus and a 56.2% decrease in immobilized YRGDS. PBFP hydrogels also demonstrated gradients in hydrogel degradation with degradation times ranging from 10–12 hours in the more crosslinked regions to 4–6 hours in less crosslinked regions. An *in vitro* model of neovascularization, composed of co-culture aggregates of endothelial and smooth muscle cells, was used to evaluate the effect of these gradients on vascular sprout formation. Aggregate invasion in gradient hydrogels occurred bi-directionally with sprout alignment observed in the direction parallel to the gradient while control hydrogels with homogeneous properties resulted in uniform invasion. In PBFP gradient hydrogels, aggregate sprout length was found to be twice as long in the direction parallel to the gradient as compared to the perpendicular direction after three weeks in culture. This directionality was found to be more prominent in gradient regions of increased stiffness, crosslinked MMP-sensitive peptide presentation, and immobilized YRGDS concentration.

## Introduction

Tissue engineering seeks to replace or restore damaged and/or diseased tissues using a combination of biocompatible, biodegradable polymer scaffolds, biofunctional molecules and cells. Although significant progress has been made with respect to engineering thin or avascular tissues, the regeneration of thicker and more metabolically demanding tissues has been hindered by the inability to promote rapid and stable neovascularization resulting in insufficient oxygen and nutrient delivery. Therefore, the continued enhancement of tissue engineered scaffolds is highly dependent on the ability to stimulate rapid and stable neovascularization within a biomaterial [1].

A variety of growth factors have been shown to stimulate neovascularization, however, the spatial presentation of these as well as other extracellular matrix (ECM) signals play a critical role in this process. Gradients of immobilized ECM molecules [2] and soluble growth factors [3] have been shown to guide vascularization. *In vivo* studies of the retina have demonstrated that gradients of secreted vascular endothelial growth factor (VEGF) help shape vascular patterns during angiogenic sprouting by guiding endothelial tip cell formation and subsequent migration [4]. Computational models have also predicted that VEGF gradients in hypoxic muscle tissue are capable of directing the formation of new vessels [5]. *In vitro* studies using rigid substrates have shown that endothelial cells align with laminin [6] and fibronectin [7] gradients, with cells adhering preferentially to regions of greater protein density. Furthermore, the presentation of simultaneous gradients of VEGF and fibronectin promote increased endothelial cell migration as compared to surface gradients of either protein alone [8].

While the use of rigid substrates has provided significant inight on the role of specific gradients on endothelial cell behavior and neovascularization on two-dimensional (2D) surfaces, the creation of gradients within three-dimensional (3D) scaffolds more accurately mimics the native physiological environment. Limited studies have investigated the effect of gradients on 3D cell behavior. The effects of gradients of VEGF and matrix composition on 3D endothelial cell invasion have been investigated in natural scaffolds such as collagen [9] and semi-interpenetrated networks of collagen and hyaluronic acid (HA) [10]. Directed endothelial cell invasion within porous collagen scaffolds has been found to occur due to gradients of immobilized VEGF [9], while endothelial cell sprout formation has been shown to be guided towards regions of decreased HA concentration in collagen-HA interpenetrating networks with observed two-fold increases in sprout length as compared to that occurring in isotropic matrix controls [10]. These studies strongly suggest that gradients of matrix cues lead to enhanced and directed endothelial cell behavior and play a critical role in inducing vascular sprout formation.

Gradients of signals and matrix properties have also been embedded within synthetic polymeric hydrogels [11]–[18]. As compared to natural biomaterials, the main advantage of using synthetic hydrogel formulations is that they offer environments for selective tuning and incorporation of biofunctional ECM signals as well as manipulation of mechanical properties enabling controlled study of cell-substrate interactions. Among the various classes of synthetic biomaterials, hydrogels of poly(ethylene glycol) diacrylate (PEGDA) have been extensively investigated as scaffolds in tissue engineering due to their tunable physical and mechanical properties. The intrinsic resistance of PEGDA scaffolds to non-specific cell adhesion and protein adsorption also provides a blank slate upon which ECM moieties can be selectively incorporated. The use of microfluidic techniques in combination with photopolymerization for the generation of stiffness gradients in PEGDA scaffolds has resulted in the preferential adhesion of macrophages in regions of increased stiffness [11] and in increased 2D endothelial cell adhesion in regions of higher immobilized concentrations of the cell adhesion ligand RGD [12]. Similar directional responses have been observed in photopolymerized PEGDA hydrogels with gradients produced by gradient makers. Smooth muscle cells have been shown to migrate towards regions of higher concentrations of immobilized basic fibroblast growth factor (bFGF) [14] and fibroblast alignment [13], [15] and directional migration has been observed [13], [16] on hydrogel surfaces with gradients of immobilized RGD.

While the above mentioned PEGDA hydrogel studies have previously reported directed cellular behavior in response to an individual type of gradient of biofunctionality or matrix stiffness, it is not entirely clear whether these targeted gradients induced spatial variations in other biomaterial properties upon hydrogel crosslinking. In a recent study, we quantified the presence of multiple types of gradients in PEGDA scaffolds where the gradients were generated using a novel photopolymerization technique (perfusion-based frontal photopolymerization (PBFP)). Specifically, the use of PBFP resulted in the generation of PEGDA hydrogels with tunable gradients of immobilized YRGDS and crosslink density and/or elastic modulus. This previous study demonstrated that simultaneous gradients in YRGDS and elastic modulus result in bi-directional fibroblast aggregate outgrowth on the surface of PEGDA hydrogels with cells spreading twice as far in the direction parallel to the gradient as compared to the perpendicular direction [18]. Although techniques for gradient generation can be used to target specific and individual gradients in synthetic scaffolds, the ability to readily translate these approaches for directing 3D cell behavior becomes a challenging task as an increased number of scaffold design variables are required in order to promote cell adhesion, proliferation, migration, and scaffold degradation.

While PEGDA hydrogels with embedded gradients offer a 3D environment for investigating and directing cell behavior, studies to date have exploited the use of these scaffolds to explore the effects of gradients on 2D cell behavior, which is not representative of the 3D *in vivo* microenvironment. To our knowledge the effects of gradients for directing 3D cell behavior within synthetic PEG hydrogel scaffolds has not been previously investigated and would provide significant insight for applications in regenerative medicine as well as physiologic processes such as neovascularization where the presence of gradients play an important role. In this study we evaluate and quantify the influence of simultaneous gradients on 3D directed cell invasion and vascularization within synthetic PEGDA hydrogels formed by PBFP. While the combination of mechanical and biochemical gradients may not be necessary for all tissue types, inclusion of these gradients holds immense potential for complex tissue regeneration [19]. PEGDA hydrogels were engineered with simultaneous gradients in elastic modulus, immobilized YRGDS cell adhesion ligands, and crosslinked proteolytically degradable matrix metalloproteinase (MMP)-sensitive domains. Due to the important role that MMP enzymes play during neovascularization, MMP-sensitive peptide domains were crosslinked within the PEGDA hydrogel network to enable hydrogel degradation by vascular MMP cell secretions and 3D cell invasion and sprout formation within the scaffolds. Using an *in vitro* neovascularization assay [20], [21], aggregates of endothelial and smooth muscle cell co-cultures were embedded within PEGDA hydrogels containing gradients of YRGDS, MMP-sensitivity, and elastic modulus. Aggregates seeded within the PEGDA gradient hydrogels exhibited bi-directional sprout alignment and invasion while those placed in PEGDA hydrogels with homogeneous distributions of biochemical cues and mechanical properties invaded uniformly in all directions. Furthermore, the degree of directionality and depth of invasion was found to be dependent on the localized gradient properties.

## Materials and Methods

### Materials

Dimethylformamide (DMF), O-benzotriazole-N,N,N’,N’-tetramethyl-uronium-hexafluoro-phosphate (HBTU), trifluoroacetic acid (TFA), Fmoc-amino acids and the Wang resin were obtained from AAPPTec (Louisville, KY). N,N-Diisopropylethylamine (DIEA), thioanisole, triisopropylsilane (TIS) and diethyl ether were obtained from Fisher Scientific (Hanover Park, IL). Piperidine, phenol, N-vinylpyrrolidone (NVP), triethanolamine (TEA) and eosin Y were obtained from Sigma Aldrich (St. Louis, MO).

### Peptide Design, Synthesis and Purification

Hydrogels were rendered sensitive to cell-mediated proteolysis by the inclusion of the MMP-sensitive peptide sequence VPMS↓MRGG [22], [23]. This peptide domain is sensitive to cleavage by numerous MMP’s including collagenase-1 (MMP-1), gelatinase A (MMP-2) and gelatinase B (MMP-9), which play a major role in ECM remodeling and are secreted by vascular cells. The sequence was modified with additional glycine (G) spacers and a c-terminal lysine (K) yielding the final sequence GGVPMS↓MRGGK to allow it to be capped on either end with a PEG acrylate group upon conjugation as described below. Cell adhesion to the otherwise inert PEGDA hydrogel was supported by the immobilization of the YRGDS cell adhesion ligand to the crosslinked network. Peptides were synthesized by solid-phase peptide synthesis based on standard Fmoc chemistry using a Focus Xi automated peptide synthesizer (AAPPTec, Louisville, KY). Amino acid coupling was carried out on a Wang resin in the presence of DIEA and HBTU (AA:HBTU:DIEA = 1∶1:2 M) with Fmoc protective groups cleaved by treatment with 20% piperidine in DMF. Peptides were cleaved from the resin in a TFA cleavage cocktail (90% TFA, 2.5% TIS, 2.5% thioanisole, 2.5% phenol and 2.5% deionized water), precipitated in cold diethyl ether and purified by reverse-phase high-performance liquid chromatography (HPLC). Synthesis was confirmed by ion trap time-of-flight (IT-TOF) mass spectroscopy (Shimadzu, Columbus, MD) and peptides were stored as a lyophilized powder at −20°C until use.

### Proteolytically Degradable PEGDA Macromer Synthesis

Synthesis of acryl-PEG_2000_-GGVPMS↓MRGGK-PEG_2000_-acryl (MW ∼ 5200 Da), which will henceforth be referred to as degradable PEGDA_5200_, was achieved by reacting both the terminal amine as well as the amine side group of the c-terminal lysine with the succinimidyl valerate ester (SVA) of an acryl-PEG_2000_-SVA (Laysan Bio Inc., Arab, AL) in 50 mM sodium bicarbonate (pH 8) at a 1∶2 mole ratio for four hours. The reaction mixture was then dialyzed for 24 hours to remove unreacted reagents, lyophilized and stored at −80°C until use.

### YRGDS PEG Macromer Synthesis

Acryl-PEG_3400_-YRGDS was synthesized by reacting the terminal amine of the cell adhesion peptide YRGDS with the SVA of an acryl-PEG_3400_-SVA (Laysan Bio Inc., Arab, AL) in 50 mM sodium bicarbonate (pH 8) at a 1.05∶1 mole ratio for four hours. The reaction mixture was then dialyzed for 24 hours to remove unreacted peptide, lyophilized and stored at −80°C until use.

### Hydrogel Precursor Solution

PEGDA hydrogels were generated from a precursor solution consisting of 7.5% (w/v) degradable PEGDA_5200_, 17.5% (w/v) PEG_3400_, 225 mM TEA, 37 mM NVP and 10 mg/mL acryl-PEG_3400_-YRGDS dissolved in deionized water (pH 8.3). The unacrylated PEG_3400_ was included to increase the density of the precursor solution and ensure that the perfused eosin solution remained buoyant at lower precursor concentrations of PEGDA to initiate the frontal polymerization process as previously described [18]. After polymerization, hydrogels were allowed to swell for 48 hours with several bath water changes to achieve equilibrium swelling and removal of the unacrylated PEG_3400._by diffusion. For cell invasion experiments, the precursor solution was sterile filtered using a 0.2 µm Teflon syringe filter.

### Hydrogel Formation via PBFP

**Figure 1:**
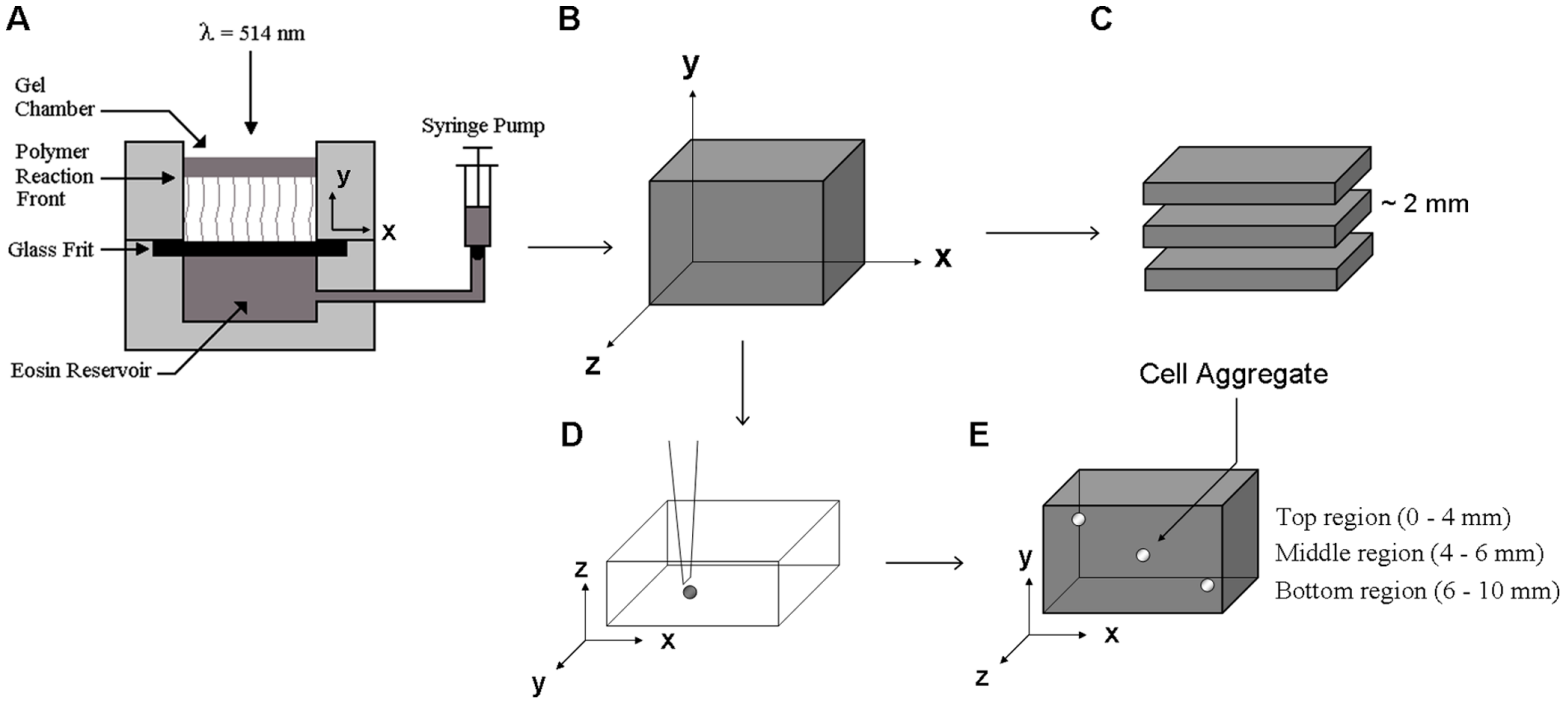
PBFP hydrogel generation and gradient evaluation. (A) Schematic of glass frit perfusion chamber and (B) resultant hydrogel. (C) Hydrogel sectioning for gradient evaluation as well as (D) aggregate seeding in hydrogels (note: hydrogels were rotated 90° to facilitate aggregate placement in the z-direction) and (E) aggregate location by region. In all cases, the gradient runs in the y-direction with the top of the gel taken as the staring point for gradient characterization.

PEGDA hydrogels were generated by PBFP as has been previously described [18]. Briefly, the precursor solution (1 mL) was loaded into a custom made chamber (Figure 1A) containing a glass frit filter disk that separated it from a reservoir of the eosin Y photoinitiator (0.1 mM), which was pressurized by a syringe pump. Photopolymerization was commenced by initialization of the syringe pump at a flow rate of 20 µL/min and exposure to 10 mW/cm^2^ visible light (λ = 514 nm) using an Argon Ion Laser (Coherent Inc., Santa Clara, CA). The buoyant eosin Y enters the chamber and rises to the surface of the precursor solution. Once a threshold eosin concentration has been reached, polymerization commences forming a polymer reaction front that propagates through the precursor solution and is fed from below by the continuous addition of eosin. Polymerization was carried out for 20 minutes to ensure full propagation of the reaction front. Control hydrogels were formed under similar conditions via bulk photopolymerization using a modified version of the chamber in which the glass frit filter disk was replaced by a solid base. Bulk hydrogels were formed through the addition of 400 µL of 0.1 mM eosin Y to the precursor solution prior to polymerization. This volume of eosin Y was equivalent to the time integral of the eosin volumetric flow rate and the precursor/eosin solution was well mixed to ensure uniform photopolymerization.

### Quantification of Elastic Modulus and Immobilized YRGDS Gradients in PBFP Degradable Hydrogels

Following polymerization, PBFP hydrogels and their bulk equivalents were extracted from the chamber and sectioned in approximately 2 mm increments along the x–z plane using an array of razor blades (Figure 1C). To neglect surface effects the upper 2 mm section of each gel was discarded and the upper surface of the subsequent section was taken as the starting point for gradient characterization. Each 2 mm section was then quantified with respect to mechanical properties and immobilized YRGDS concentration.

Mechanical properties of PBFP hydrogels, as well as their bulk equivalents, were quantified using compression experiments. Prior to mechanical testing, hydrogel sections were swollen for 48 hours with several bath water changes. Hydrogel sections were then loaded onto a TA RSA3 mechanical tester (TA Instruments, New Castle, DE) controlled by TA Orchestrator software. Samples were compressed at a rate of −0.5 mm/min to obtain a stress *vs.* strain curve. The elastic modulus of the hydrogel was taken as the slope of the linear region (<10% strain, r^2^>0.95) of the stress vs. strain curve as has been previously reported [24], [25].

Spatial incorporation of acryl-PEG_3400_-YRGDS into the hydrogel network was quantified by radiolabeling the tyrosine (Y) residue with ^125^I prior to gel formation [18], [26]. YRGDS was radiolabeled with ^125^I according to previously reported techniques [18], conjugated to a mono-acrylated PEG as outlined above and stored at −80°C in a lead lined container until use. To track the spatial incorporation of the YRGDS adhesion ligand in PBFP hydrogels and in bulk polymerized hydrogel controls, radiolabeled acryl-PEG_3400_-YRGDS was added to the precursor in an amount not exceeding 20 wt % of the total peptide. Hydrogels were then polymerized and sectioned as outlined above. Hydrogel sections were swollen in deionized water for 48 hours with a minimum of two bath water changes to allow for diffusion of unreacted acryl-PEG_3400_-YRGDS. Following washing, the radioactivity of each section was determined using a Packard Cobra II gamma counter (PerkinElmer Health Sciences Inc., Shelton, CT) and the concentration of immobilized YRGDS in each sectioned quantified based on the specific activity of the radiolabelled YRGDS and the final hydrogel volume.

### Quantification of Gradients of Hydrogel Degradation

Spatial variations in degradation time as a function of the gradient in MMP-sensitive crosslinks was evaluated by incubating the hydrogel sections in a fixed concentration of an interstitial collagenase (Collagenase-1A, MMP-1). PBFP gradient hydrogels and their bulk equivalents were sectioned as outlined above and swollen in deionized water for 36 hours before being transferred to pre-weighed tubes and incubated in 10 mM HEPES buffered saline (HBS; 10 mM HEPES sodium salt and 137 mM NaCl) with 1 mM CaCl_2_ (pH 7.4) overnight. Collagenase-1A (Sigma Aldrich) at a concentration of 1 µg/mL was added to the hydrogel sections and the swollen weight of each section was recorded every two hours until complete hydrogel degradation was achieved. Fresh enzyme suspended in 10 mM HBS with 1 mM CaCl_2_ (pH 7.4) was added following each measurement. In order to ensure that hydrogel degradation was due to the specific action of MMP-1, control studies were performed with bulk hydrogels placed in a buffer solution lacking enzyme.

### Cell Culture and Vascular Aggregate Formation

Human umbilical vein endothelial cells (HUVECs) were obtained from Lonza (Walkersville, MD) and maintained in endothelial growth media (EGM Bullet kit, Lonza) containing 2% fetal bovine serum (FBS), human epidermal growth factor (hEGF), hydrocortisone, bovine brain extract (BBE), ascorbic acid and gentamicin/amphotericin (GA). Human umbilical artery smooth muscle cells (HUASMCs) were obtained from Lonza and maintained in smooth muscle growth media (SmGM-2 bullet kit, Lonza) supplemented with 2% FBS, hEGF, human fibroblastic growth factor (hFGF), insulin and GA. Cells were grown to 80–90% confluency and collected by incubation with Trypsin for 5 minutes followed by centrifugation for 10 minutes at 200 g. Cell aggregates composed of 50% HUVEC and 50% HUASMC were formed by suspending both cells types (5000 cells/well) in EGM culture media containing 0.24% (w/v) methyl cellulose (Sigma-Aldrich) in round-bottom, low binding 96 well-plates (Fisher Scientific). Cells were incubated overnight at 37°C and 5% CO_2_. Once the aggregates had formed, they were removed from the wells and washed in culture media prior to monitoring cell behavior within PEGDA hydrogels as described below. In order to visualize the presence of endothelial and smooth muscle cell interactions within the vascular sprouts, each cell type was fluorescently labeled using different stains prior to aggregate formation. HUASMCs and HUVECS were fluorescently labeled using PKH26 and CellVue® Claret cell membrane labels (Sigma Aldrich), respectively. Following aggregate formation and placement within PEGDA hydrogels, fluorescently labeled sprout invasion was imaged daily using dual channel confocal microscopy (Carl Zeiss Inc., Göttingen, Germany) until the fluorescent signal diminished below detectable levels.

### Analysis of 3D Vascular Sprout Formation in PBFP Degradable Hydrogels

**Figure 2:**
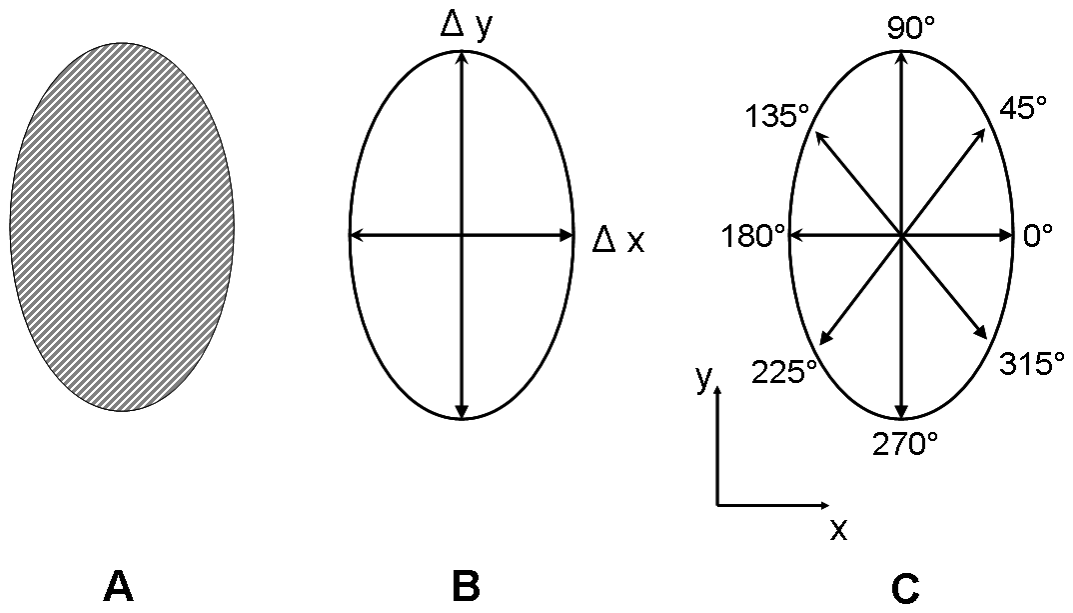
Quantification of aggregate invasion. Schematic representation of the methods used to quantify aggregate invasion by (A) total projected area as well as directional vascular sprout formation via (B) anisotropy index and (C) sprout length by angle.

Following polymerization, hydrogels were swollen for 36 hours in 10 mM HBS (pH 7.4) followed by 12 hours in endothelial basal media in preparation for aggregate implantation. The schematic in Figure 1B illustrates that PBFP results in gradients that run in the y-direction. For cellular studies, the hydrogels were laid flat with the x-y plane running in the horizontal direction so that the aggregates could be positioned in different regions of the gradient (running in the y-direction). Aggregates were gently placed inside the gels using a micropipette tip (Figure 1D) with aggregate injection performed along the z-axis. Aggregates were situated in the upper (0–4 mm from top), middle (4–6 mm from top), and lower regions (6–10 mm from top) of PBFP gradient hydrogels (Figure 1E) to evaluate the effects of different gradient properties on cell invasion. Aggregates were also seeded within the middle region of bulk polymerized control gels with homogeneously distributed properties. Eight aggregates per region per gel type were seeded with a maximum of two aggregates per region spaced at least 8 mm apart (n = 4 gels/type). Aggregates were imaged along their central axis weekly for three weeks using an Axiovert 200 inverted microscope (Carl Zeiss Inc.) with cell culture media (EGM) changed every other day. Phase contrast images were used to quantify aggregate invasion over time in terms of projected aggregate area (Figure 2A), the aggregate diameter in the direction perpendicular (x-direction) and parallel (y-direction) to the gradient, the anisotropy index (Δy/Δx; Figure 2B), and sprout length by angle (Figure 2C) using Axiovision 4.2 image analysis software (Carl Zeiss Inc., Göttingen, Germany). To better visualize 3D invasion, aggregates were formalin fixed after three weeks of culture and stained for F-actin using Alexa Fluor 546-phalloidin (5 units/mL in PBS; Invitrogen, Eugene, OR). The stained aggregates were then imaged using LSM confocal microscopy (Carl Zeiss) to produce a z-stack of images that could be flattened to obtain a 3D rendering of sprout invasion.

### Statistical Analysis

Comparisons between various conditions are expressed as mean ± standard deviation. Statistical analysis was evaluated using either one- or two-way ANOVA followed by a Tukey’s post-test (SigmaStat 3.5, San Jose, CA) with p<0.05 considered significant. Normality was confirmed prior to running ANOVA by a Kolmogorov-Smirnov normality test with normality confirmed by p>0.05.

## Results

### Quantification of Spatial Variations in ECM Properties in PBFP Gradient Hydrogels

**Figure 3:**
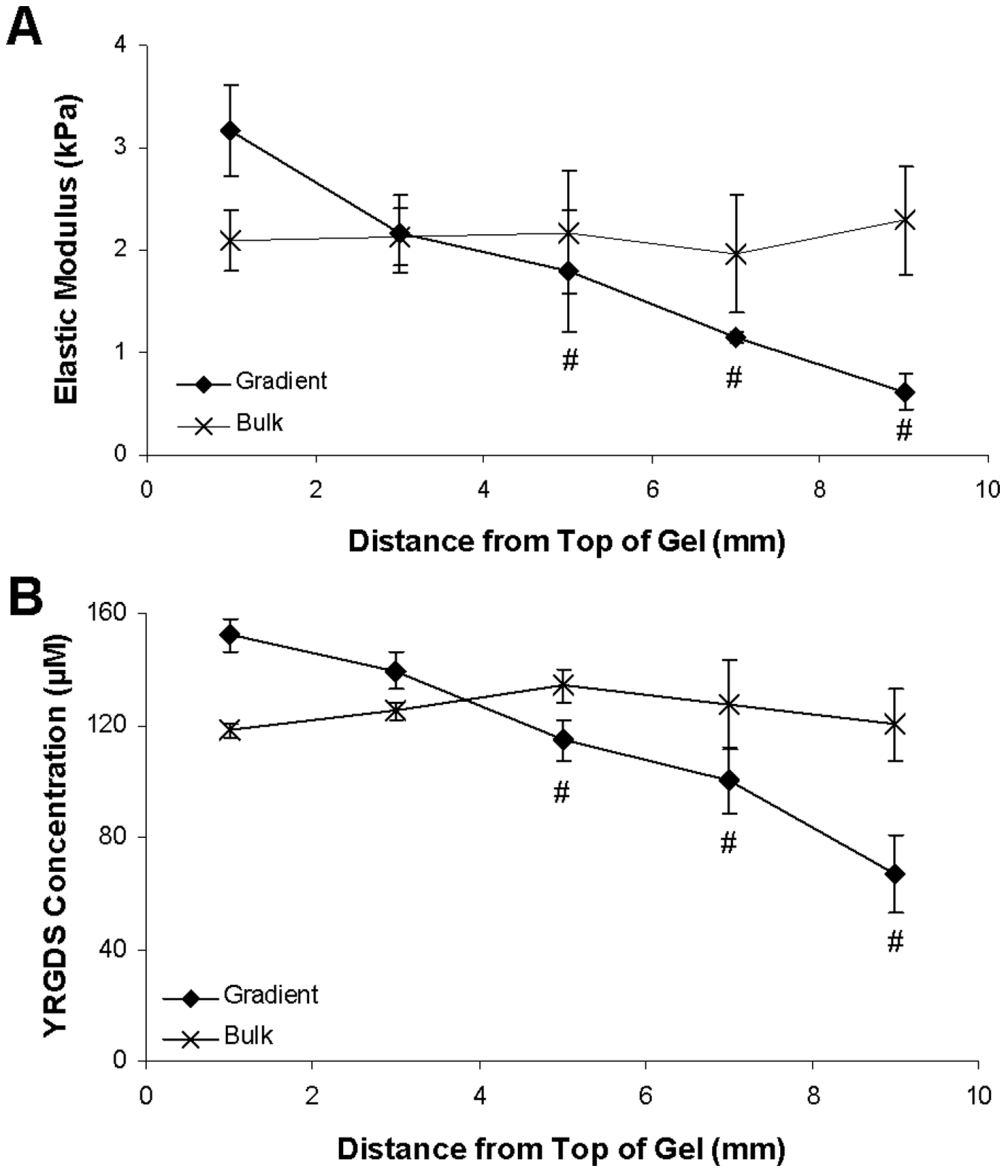
Quantification of hydrogel mechanical and biofunctional gradients. Spatial variations of (A) hydrogel elastic modulus and (B) immobilized concentrations of YRGDS in MMP-sensitive PBFP gradient hydrogels as well as equivalent bulk control gels (# = p<0.05 as compared to top section (0–2 mm) of gel; n = 3). Error bars represent ± standard deviation.

Spatial variations in hydrogel elastic modulus and immobilized concentrations of the YRGDS cell adhesion ligand were evaluated using compression testing and radiolabeling experiments, respectively. As shown in Figure 3A, the elastic modulus of gradient hydrogels decreased 80.4% from 3.17 to 0.62 kPa over a span of 10 mm (∼250 Pa/mm). This decrease was found to be statistically significant when comparing the uppermost section of the gel to the third (4–6 mm), fourth (6–8 mm), and fifth (8–10 mm) hydrogel sections. In contrast, bulk polymerized hydrogels exhibited a uniform elastic modulus of approximately 2.13 kPa with slight variations determined not to be statistically significant (p = 0.941). Similar trends were seen for immobilized concentrations of YRGDS (Figure 3B). PBFP gradient hydrogels had a 56.2% decrease in YRGDS concentration from 152.2 to 66.7 µM over 10 mm (∼8.6 µM/mm) while bulk hydrogels had an average immobilized YRGDS concentration of 124.9 µM with fluctuations that were determined to be not statistically significant (p = 0.326). The observed decrease in YRGDS concentration in gradient hydrogels was determined to be statistically significant when comparing the uppermost hydrogel section to the third (4–6 mm), fourth (6–8 mm), and fifth (8–10 mm) sections. The elastic modulus and immobilized YRGDS concentration of bulk polymerized hydrogels fell within the range of values of gradient scaffolds.

**Figure 4:**
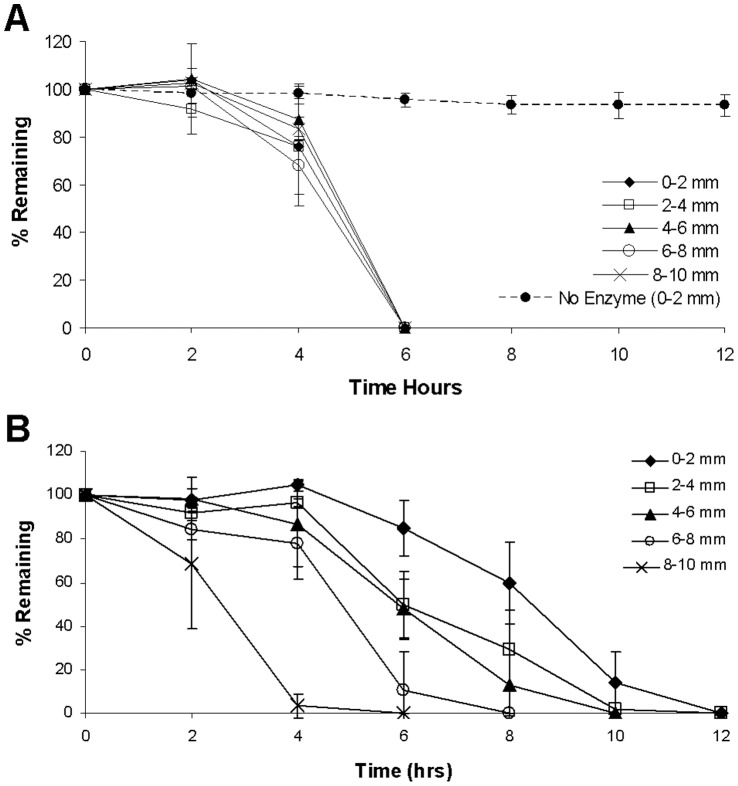
Quantification of hydrogel degradation kinetics. Degradation kinetics of hydrogel sections taken from different regions of (A) bulk polymerized control gels and (B) PBFP gradient hydrogels when exposed to 1 µg/mL collagenase-1A (MMP-1) (n = 3). Error bars represent ± standard deviation.

PBFP has been previously shown to result in gradients of crosslinking [18]. In the present study, hydrogel crosslinking is exclusively induced from PEGDA macromers containing an MMP-sensitive domain between the terminal acrylate groups resulting in proteolytically degradable crosslinks upon gelation. This suggests that the hydrogels formed using this technique will result in a gradient in MMP-sensitivity and degradation which may influence the rate at which cells at the leading edge of a vascular sprout can invade the scaffold. To quantify gradients in degradation via proteolysis, degradation experiments were conducted on both gradient and bulk hydrogel controls. Spatial gradients in degradation were quantified by sectioning hydrogels in 2 mm increments and obtaining their respective times for complete degradation following MMP-1 incubations as described above. As expected, all bulk hydrogel sections displayed a similar degradation time of six hours when exposed to a 1 µg/mL solution of collagenase-1A (Figure 4A). Control studies of bulk hydrogels placed in solutions that did not contain collagenase-1A demonstrated no degradation throughout the time course of the experiment indicating that gel degradation was due to the enzymatic action of MMP-1. In contrast, hydrogel sections obtained from PBFP hydrogels exhibited varying degradation profiles and a range of degradation times with the highest degradation time occurring in the more crosslinked and stiffer region (0–2 mm) with progressive decreases in degradation time noted in less crosslinked and softer regions of the hydrogel. As shown in Figure 4B, the top section (0–2 mm) required 12 hours to reach complete degradation while middle regions (4–6 mm) degraded fully within 8–10 hrs and bottom sections (8–10 mm) degraded within 4–6 hrs. Since the moles of enzyme present were constant in each sample and each mole of enzyme can only degrade a fixed number of proteolytic domains in a given time, this suggests there were roughly 2–3 more MMP-sensitive crosslinks present in the top or more crosslinked hydrogel regions as compared to the less crosslinked bottom regions.

### Quantification of 3D Vascular Sprout Formation in PBFP Degradable Hydrogels

**Figure 5:**
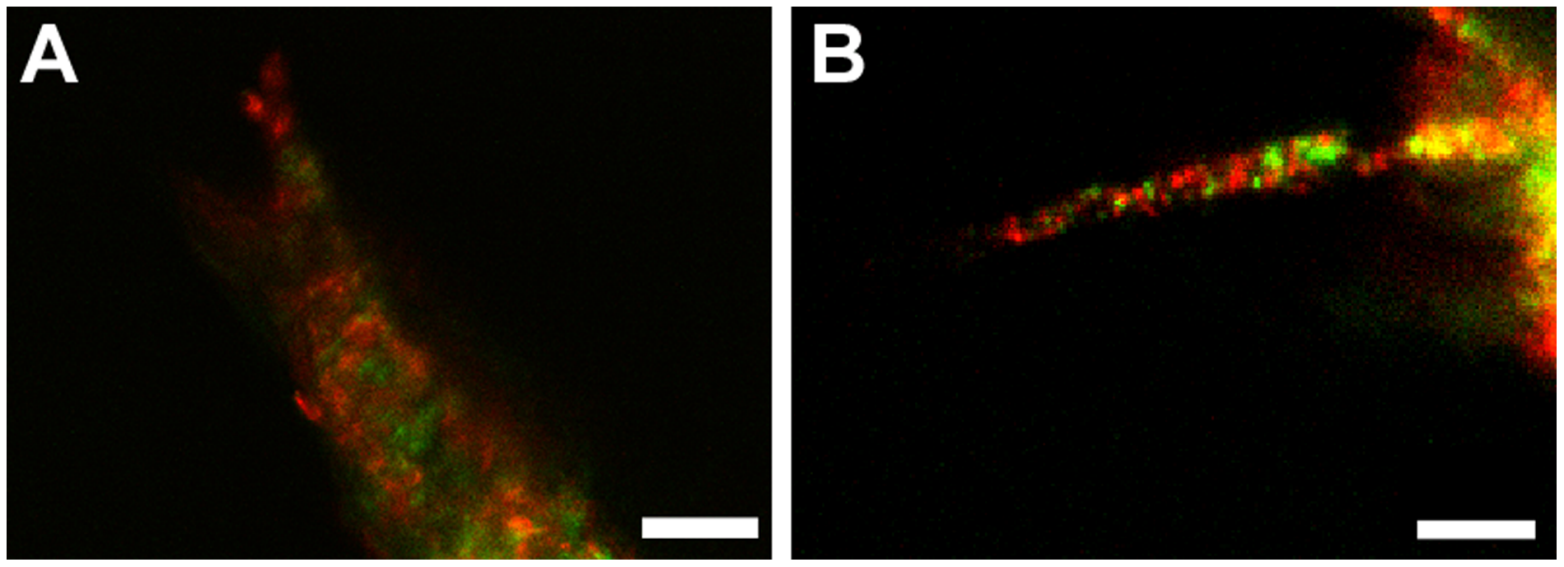
Coordinated cellular interactions of vascular sprout invasion within PEGDA hydrogels. Confocal images taken using 20× magnification at (A) 24 and (B) 48 hours post-aggregate placement in hydrogels. Fluorescently labeled endothelial cells appear in red and smooth muscle cells appear in green (scalebar = 50 µm).

Vascular sprout formation within MMP-sensitive PEGDA bulk and gradient hydrogels was achieved using a previously established co-culture sprouting model of neovascularization composed of HUVECs and HUASMCs [21]. *In vivo*, vascular sprout formation and vessel stabilization within the extracellular matrix involves coordinated cellular interactions between endothelial and mural cells such as smooth muscle cells which are critical to vessel assembly [21], [27], [28]. Therefore, the 3D *in vitro* co-culture model was chosen to more accurately recapitulate the coordinated cell processes of *in vivo* vascular sprout invasion. Previous results have shown that both cell types contribute to formation and stabilization of networks in this model [21]. In order to verify these results, HUVECS and HUASMCs were labeled with different fluorescent labels as previously described prior to aggregate formation. Dual stained aggregates were then embedded in PEGDA hydrogels and imaged daily using confocal microscopy. Figure 5 shows that vascular sprouts emanating from the aggregate body are a result of coordinated interactions between the two cell types. Furthermore, HUVECs (red) are seen at the tip of the sprout leading vascular invasion (Figure 5A).

**Figure 6:**
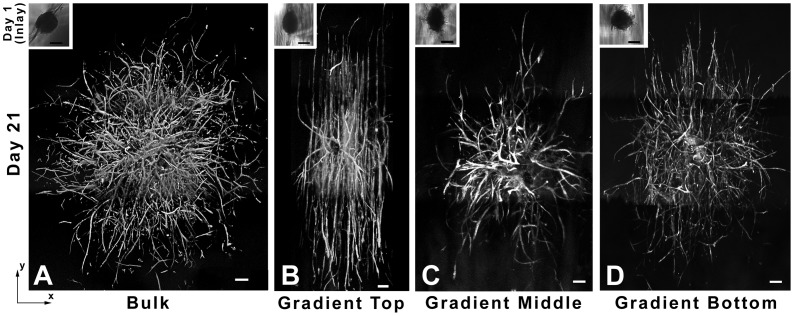
Vascular sprout invasion within PEGDA hydrogels. Flattened 3D mosaic renderings of co-culture aggregates seeded in (A) bulk control hydrogels as well as in the (B) top, (C) middle, (D) and bottom regions of PBFP gradient hydrogels. Aggregates were fixed after 21 days in culture and stained for F-actin (Note: the gradient runs in the y-direction in B, C, and D with elastic modulus and YRGDS concentration increasing in the direction of the top of the image; scalebar = 200 µm).

**Figure 7:**
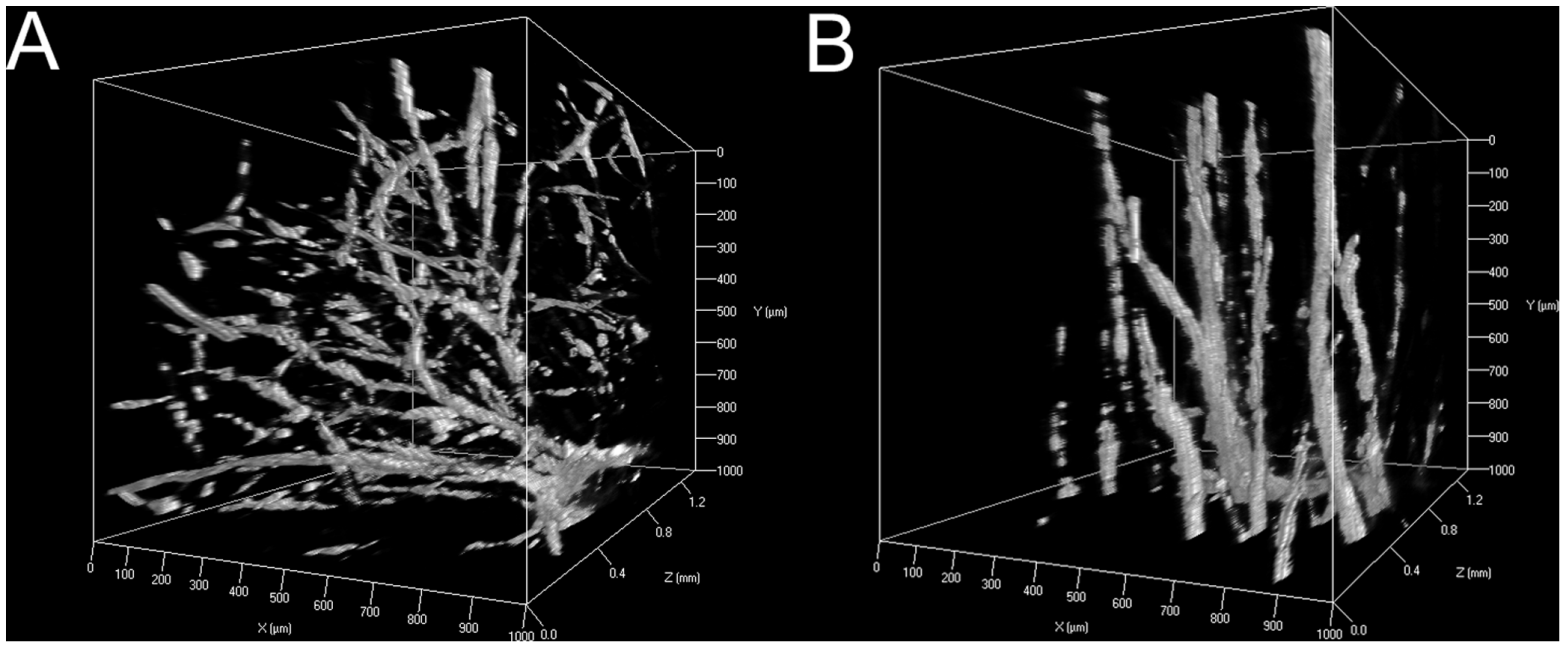
Three-dimensional vascular sprout invasion in PEG Scaffolds. 3D fluorescent image reconstructions of vascular sprout invasion in (A) bulk control gels and (B) PBFP gradient hydrogels taken at 10× magnification (Note: the gradient runs in the y-direction in B).

In order to quantify cell invasion and sprout formation as a function of gradient properties, co-culture aggregates of endothelial and smooth muscle cells were seeded within the top (0–4 mm), middle (4–6 mm), and bottom (6–10 mm) regions of PBFP gradient hydrogels as well as within bulk polymerized control gels. Aggregates seeded in the top region of PBFP gradient gels were exposed to an elastic modulus ranging from 3.17 to 2.16 kPa and a YRGDS concentration of 152.2 to 139.4 µM while aggregates in the middle and bottom regions of the gradient gels were subjected to an elastic modulus ranging from 2.16 to 1.15 kPa and 1.15 to 0.62 kPa, respectively, as well as a YRGDS concentration of 139.4 to 99.9 µM and 99.9 to 66.7 µM, respectively. Aggregates seeded in the top hydrogel regions were also exposed to a higher concentration of MMP-sensitive crosslinks than those seeded in lower less crosslinked regions. In bulk hydrogel controls, aggregates were exposed to uniform concentrations of YRGDS (124.9 µM) and MMP-sensitive crosslinks as well as a homogeneous elastic modulus (2.13 kPa). The properties of bulk hydrogel controls were similar to those of the middle region of the PBFP gradient hydrogels. Sprout invasion and directionality was visualized by F-actin staining after 3 weeks in culture (Figure 6). As shown in Figure 6A, aggregates seeded in bulk control gels resulted in uniform and random sprout invasion in all directions. In contrast, aggregates seeded in the top of the gradient hydrogels exhibited sprouts that curved and invaded bi-directionally both up and down the gradient (Figure 6B). Aggregates seeded in the middle and bottom regions of PBFP hydrogels exhibited a lower degree of sprout alignment as compared to those within the upper regions of PBFP scaffolds (Figures 6C and 6D). In the bottom and least crosslinked region of the gradient hydrogels directional sprouting occurred up the gradient with more randomly oriented sprout formation resembling that observed in the isotropic scaffolds occurring down the gradient (Figure 6D). Sprout invasion and directionality was also confirmed to occur in 3D within the scaffolds. Figure 7 displays 3D renderings of localized sprout invasion in bulk control hydrogels (Figure 7A) as well as in the top region of PBFP gradient hydrogels (Figure 7B). As seen by these images, aggregates seeded in bulk control hydrogels exhibit randomly oriented sprouts emanating in all directions from the aggregate body while aggregates subjected to a gradient respond directionally with sprouts aligning parallel to the gradient.

**Figure 8:**
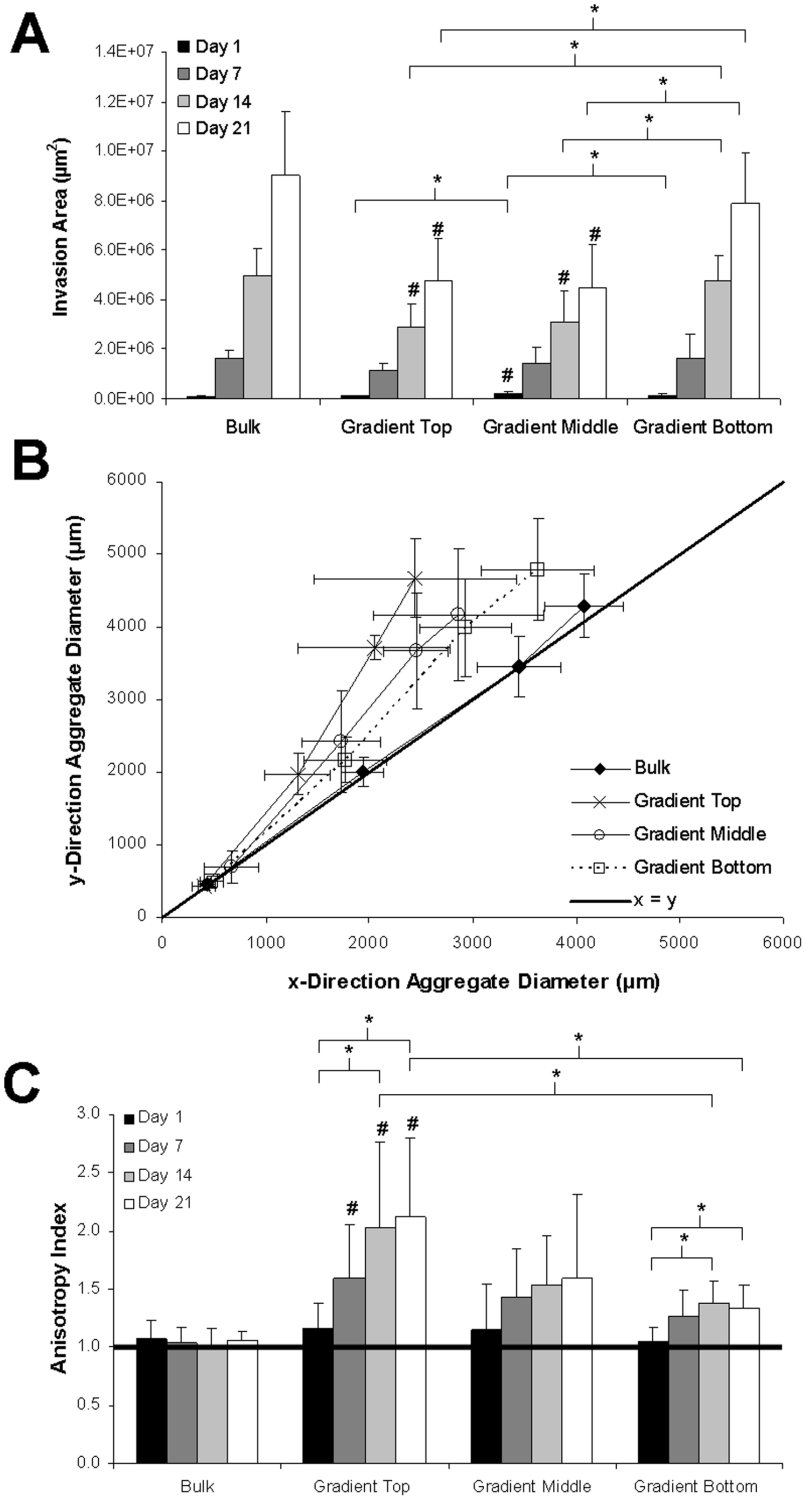
Characterization of vascular sprout invasion. (A) Total projected area, (B) quantile-quantile (Q–Q) plot of x- and y-direction aggregate diameters, and (C) anisotropy index of co-culture aggregates seeded within the top, middle, and bottom regions of PBFP gradient hydrogels as well as in bulk control gels over time (n = 8; * = p<0.05 (significance between time points omitted in (A) for clarity); ^#^ = significant difference (p<0.05) from bulk control at same time point). Error bars represent ± standard deviation.

Cell invasion within the various regions of the gradient hydrogels as well as within the isotropic bulk controls was monitored over time by quantifying the total projected area of the aggregate and resulting sprout formation. As shown in Figure 8A, statistically significant differences in aggregate invasion were observed in the presence of the gradient. By day 14, aggregates seeded in the top and middle region of gradient scaffolds invaded significantly less than those seeded in both the bulk controls as well as in the bottom region of the gradient gels. By day 21, the total invasion area of aggregates seeded in the top and middle of the gradient gels was about half of the area of aggregates located in bulk control gels and three quarters of the area of the aggregates in the bottom region of the gradient gels. Similar trends in aggregate area were observed when comparing invasion areas of the bottom region of gradient hydrogels to the bulk control with a 25% decrease in invasion occurring in the gradient hydrogels, even though this difference was not found to be statistically significant. The observed decreases in total projected area in the gradient hydrogels (Figure 8A) are due to the fact that the cells are only growing along the gradient direction as opposed to growing in all directions as in the case of the isotropic scaffolds (Figure 6A).

Aggregate sprout orientation in response to the gradients generated was evaluated by quantifying the x- and y-direction aggregate diameters (Figure 8B) as well as the anisotropy index (Figure 8C) over time. As shown by the Q–Q plot in Figure 8B, aggregates seeded in PBFP gradient hydrogels deviate from the equality reference line towards the y-axis over time while aggregates seeded in bulk control gels invade uniformly and lie on the reference line. This indicates that aggregates seeded in gradient hydrogels favor growth in the y-direction, or in the direction of the gradient. Furthermore, decreases in the distance of aggregate placement from the top-end of the gradient result in increased directional sprout invasion. In terms of anisotropy index, aggregates seeded in the top regions of PBFP gradient hydrogels invaded roughly twice as far in the direction of the gradient as compared to the perpendicular direction by day 21 (anisotropy index = 2.12±0.68) with directional invasion becoming significant by day 14. The directionality of sprout invasion in the top region was statistically significant as compared to that of isotropic bulk controls, for which aggregates exhibited an anisotropy index of one across all time points indicative of uniform sprout invasion in all directions. Aggregates seeded in the middle and bottom region of the gradient also invaded directionally (anisotropy index = 1.59±0.72 and 1.33±0.20, respectively) although the degree of directionality by day 21 was not as prominent as in the case when the aggregates were placed closer to the top of the gradient. Overall, the degree of directionality decreased with aggregate placement further from the top of the gradient.

**Figure 9:**
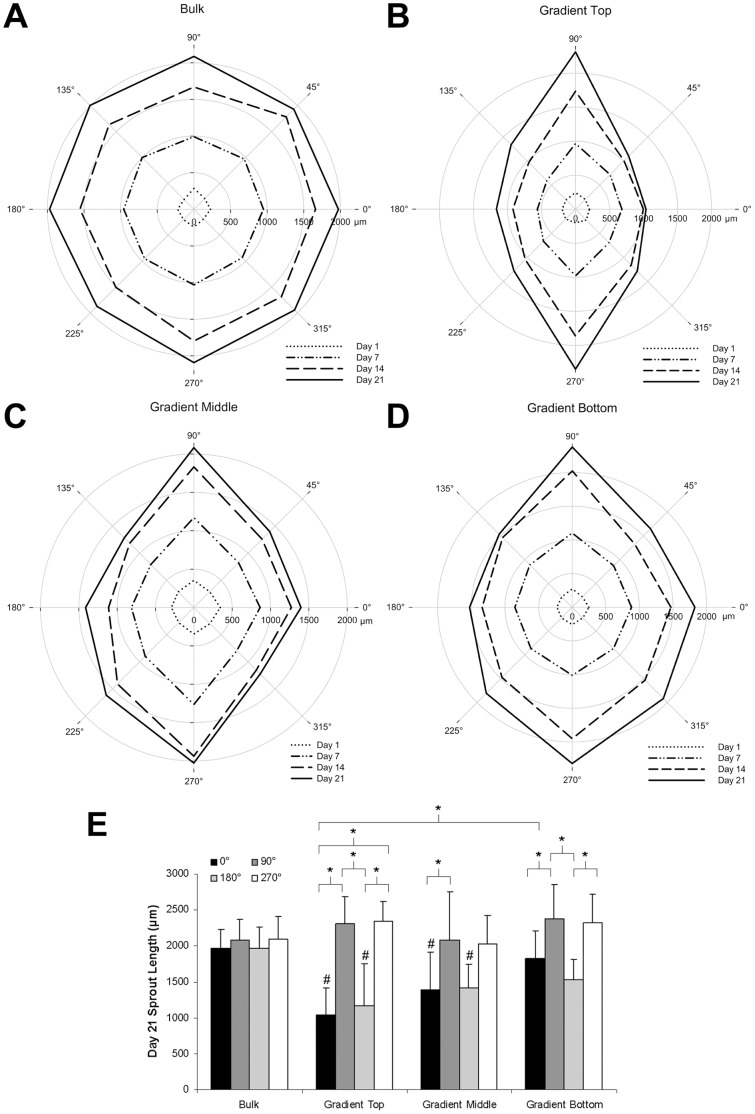
Quantification of vascular sprout length. (A-D) Average sprout length by angle of co-culture aggregates seeded within bulk control gels (A) as well as the top, middle, and bottom regions of PBFP gradient hydrogels (B-D) over three weeks (Note: gradients run from regions of high elastic modulus and YRGDS concentration at 90° to regions of lower elastic modulus and YRGDS concentration at 270°). (E) Day 21 sprout length as a function of location and gel type at 0°, 90°, 180° and 270° (n = 8; * = p<0.05; # = significant difference (p<0.05) from bulk control at same time point). Error bars represent ± standard deviation.

To further characterize the directionality of vascular sprouting, sprout length was quantified as a function of angle over time. Similar to the results observed for anisotropy index, the average sprout length for aggregates seeded in bulk control gels (Figure 9A) was equivalent in all directions at a given time point. However, aggregates seeded in the top (Figure 9B), middle (Figure 9C), and bottom (Figure 9D) regions of gradient hydrogels exhibited significantly increased sprout length at 90° and 270°, or both up and down the gradient, as compared to the perpendicular directions (0° and 180°). Additionally, aggregates placed in the middle and bottom regions of PBFP hydrogels demonstrated a longer sprout length at 0° and 180°, as compared to aggregates placed within the upper regions of the gradient, indicating that the directionality of the vascular sprouts is not as pronounced when the aggregates are placed further from the top of the gradient. These differences are summarized in Figure 9E, which displays the average-sprout length by angle in the different regions of the PBFP hydrogels as well as in bulk control gels at day 21. Aggregates seeded in both the top and bottom regions of the gradient have statistically significant differences in sprout length in the direction of the gradient (90° and 270°) as compared to the perpendicular directions (0° and 180°) while those seeded in the middle region exhibit similar characteristics. In the top region of the gradient, vascular sprouts reach a length of 2311±374 µm up the gradient and 2348±267 µm down the gradient while averaging 1100±481 µm in perpendicular directions. In the middle region of the gradient, vascular sprouts reach a length of 2082±670 µm up the gradient and 2025±391 µm down the gradient while averaging 1403±423 µm in the perpendicular direction. Vascular sprouts in the bottom region of the gradient reach a length of 2378±471 µm up the gradient, 2320±396 µm down the gradient and 1675±337 µm in the perpendicular direction. Overall, sprout length in the direction of the gradient remains fairly constant across the various regions while sprout length in the direction perpendicular to the gradient increases step-wise as cellular aggregates are placed further from the top of the gradient. In contrast, the average sprout length of aggregates seeded in bulk control gels remained constant at all angles at approximately 2031±285 µm. Interestingly, by day 21, the maximum vascular sprout length was observed along the gradient in hydrogels formed via PBFP. Although this difference was not found to be statistically significant as compared to bulk control gels, on average, the sprout length in the direction of the gradient was approximately 10% longer than that observed in bulk hydrogel controls.

## Discussion

The dimensions of tissue engineered constructs are dependent on the extent to which vessels can be stimulated to form within the implant. Therefore, the continued enhancement of tissue engineered scaffolds relies on the ability of the construct to stimulate rapid and stable neovascularization [1]. While the inclusion of growth factors has been shown to stimulate neovascularization [1], [29]–[33], the spatial presentation of these as well as other ECM signals plays a critical role in this process. In this study, PBFP was used to create PEGDA hydrogels with simultaneous gradients in elastic modulus, YRGDS adhesion ligands, and MMP-sensitive degradation sites in order to investigate spatial variations of these matrix cues on stimulating 3D directed neovascularization. Vascular cells seeded within the PEGDA gradient hydrogels resulted in bi-directional sprout alignment and invasion while those placed in equivalent PEGDA hydrogels with homogeneous distributions of biochemical cues and mechanical properties invaded uniformly in all directions. The degree of directionality was found to be dependent on the localized gradient properties with sprout alignment increasing in regions with a higher elastic modulus as well as higher concentrations of immobilized YRGDS and crosslinked MMP-sensitive domains.

The use of crosslinked MMP-sensitive sequences in PEG hydrogels with homogeneously distributed biofunctionality and mechanical properties has been previously explored due to the important role of MMP enzymes during neovascularization [29], [34]–[38]. In the present study, we demonstrate that spatial gradients of MMP-sensitivity and proteolytic degradation induce directed neovascularization within PEGDA hydrogels. Specifically, PEGDA hydrogels were rendered sensitive to cell-mediated proteolysis by synthesizing PEGDA macromers containing an MMP-sensitive peptide sequence (GGVPMS↓MRGGK) within the end terminal acrylate groups, enabling the immobilization of MMP-sensitivity within the PEGDA hydrogel crosslinks. This specific peptide sequence was selected because it can be readily cleaved by a variety of MMPs secreted by vascular cells and since it has been previously shown to exhibit higher catalytic activity, rapid degradation by MMP-1 and MMP-2 enzymes, enhanced cell proliferation, and invasion in the *ex vivo* chick aortic outgrowth assay as compared to other MMP-sensitive substrates [22], [23]. Quantification of hydrogel degradation kinetics revealed that PBFP scaffolds exhibited a range of degradation times with the uppermost and more crosslinked hydrogel regions requiring 2–3 times longer to degrade than the less crosslinked bottom regions (Figure 4B). Due to the fact that vascular sprout length in the direction of the gradient was found to be similar to the top, middle, and bottom regions (Figure 9E) of gradient hydrogels, increases in the presentation of MMP-sensitive crosslinks does not appear to hinder cell invasion suggesting that a higher concentration of MMP-sensitive domains may up-regulate the secretion of MMPs by vascular cells.

The effects of the presented gradients on vascular sprout formation in MMP-sensitive PEGDA hydrogels were evaluated using a previously established *in vitro* model of neovascularization based on the co-culture of endothelial and smooth muscle cells [20], [21]. Co-cultures of these cell types have been shown to promote rapid vascularization of tissue-engineered vascular grafts [39]. Furthermore, smooth muscle cells secrete numerous ECM components, including collagen, elastin, and proteoglycans, which support endothelial cell adhesion, invasion, and vascular sprout formation. Fibrillar adhesions between endothelial cells and smooth muscle cells may also alter internal signaling pathways promoting the expression of genes necessary for the formation of a stable vasculature [40]. In addition, the interactions between endothelial and smooth muscle cells up-regulate the secretion of MMPs that play a major role in remodeling of the ECM [22], [23].

Previous studies of endothelial cell motility on rigid surfaces have shown that simultaneous gradients of signals, more specifically VEGF and fibronectin, have resulted in enhanced and directional 2D EC motility as compared to surfaces with either factor alone [8]. In this study, we show that simultaneous gradients of elastic modulus, cell adhesion ligands of YRGDS, and MMP-sensitivity, result in 3D directional neovascularization as compared to hydrogel scaffolds with uniformly distributed signals and properties in the absence of exogenous growth factors. Vascular sprout formation in the presence of these combined gradients was quantified by embedding co-culture aggregates of endothelial and smooth muscle cells in various regions of PBFP gradient hydrogels as well as in bulk control gels. In order to demonstrate that the observed directionality of sprout invasion was a result of the embedded gradients, cellular aggregates were similarly placed inside bulk control hydrogels with isotropic properties. The isotropic control scaffolds exhibited uniform immobilized RGD concentration, elastic modulus, and degradation time with values that lied in between the low and high-end values of the gradient scaffolds. Figure 6C, shows that aggregates placed in bulk control gels exhibited uniform invasion with tortuous sprouts randomly emanating from the aggregate body throughout the 3D space. In contrast, co-culture aggregates embedded within PBFP gradient hydrogels exhibited a bi-directional response with vascular sprouts invading towards regions of higher elastic modulus, immobilized YRGDS, and sucesptibility to MMP degradation as well as in regions of lower modulus and decreased concententrations of these ECM signals. Since the scaffolds formed by PBFP contain gradients of crosslink denisty it is also possible that the differential diffusion of proteins may also play a role in the observed directional cellular reponse, however, further studies are required to confirm this phoenonemon. The observed bidirectional response in the presented study was unexpected and has yet to be fully understood; however, a similar bidirectional response has been observed in previous studies with endothelial cell spheroids placed in collagen-HA interpenetrating network scaffolds containing a gradient in HA [10]. Two-dimensional studies on PEG scaffolds have also demonstrated that cells are capable of detecting and aligning with a gradient [12]–[16]; however, the underlying mechanism responsible for cell alignment was not investigated. It is likely that the actin cytoskeleton of these cells is polarized by the gradient in adhesion sites and matrix rigidity. Studies using rigid substrates have shown that the lateral spacing of integrin ligands influences focal adhesion assembly [41] and that anisotropy of the cell microenvironment can govern internal cellular organization and polarity [42]. Since the directionality of cell motility is dictated by focal adhesion positioning [43], it is likely that cells are limited to migration patterns running parallel to the gradient. Furthermore, matrix rigidity may influence the observed cell migratory patterns as cell-substrate mechanical interactions are central to the regulation of focal adhesion assembly [44]. The strength of integrin-cytoskeleton linkages is also dependent on matrix rigidity and can serve as a durotactic guidance cue [45]. It has also been shown that if focal adhesions are evenly distributed, the cytoskeleton, and therefore the cell, orients randomly [43]. This may explain the lack of directionality observed in cells seeded in bulk polymerized hydrogel controls with homogeneous distributions of extracellular matrix cues.

Based on the data shown in Figure 8A, it appears as if the presence of the gradient hinders cell invasion as the invasion area of aggregates seeded in gradient hydrogels is statistically less than those seeded in bulk control gels. Furthermore, the decrease in invasion area is more pronounced at the top end of the gradient, which is consistent with previous studies concluding that increases in matrix stiffness act as a barrier for cells cultured in 3D [46]. However, this observation is misleading since the cells are exposed to anisotropic conditions and sprout length along the gradient is about 10% longer than the the average sprout length observed in bulk hydrogels (Figure 9E). While these results appear to be contradictory, the directionality of invasion in PBFP gradient hydrogels results in an eliptical invasion area whereas the invasion area in bulk hydrogel controls yields a circular footprint. While the overall sprout length was found to be shorter in the isotropic control scaffolds, the invasion area of the circular footprint is greater than that of the eliptical invasion area observed in the gradient scaffolds. Furthermore, sprouts within the gradient hydrogels exhibit less tortuosity as compared to those seen in bulk hydrogel controls. If the tortuosity of the sprouts is considered, then the overall sprout invasion depth in bulk hydrogels would likely be equivalent to that seen in gradient hydrogels. Therefore, in this study the presence of the gradient does not appear to inhibit or enhance overall sprout invasion but rather stimulates directional vascular sprout formation.

Vascular sprout invasion and alignment were found to be dependent on the gradient hydrogel properties to which cells were exposed. Aggregates located in the upper, more crosslinked region of the gradient hydrogels exhibited a much greater degree of directionality (Figure 6B and 9B) than those located in lower, less crosslinked regions (Figure 6C–D and 9C–D). Although the magnitude of the gradient is relatively constant across the entire gel, the top end of the scaffold is more crosslinked, exhibits a higher elastic modulus,and contains higher immobilized concentrations of YRGDS and MMP-sensitive peptides (Figures 3 and 4B). The increased concentration of the immobilized YRGDS cell adhesion ligand and the MMP-sensitive peptide domains most likely allows invading cells to more readily detect and orient themselves with the gradient. In the bottom region of the gradient, vascular sprouts show alignment up the gradient with less vascular sprout alignment seen in the opposite direction (Figure 6F). This response may be due to the lower concentrations of YRGDS and MMP-sensitive domains that are present in the bottom regions of the gradient hydrogels, which may potentially prevent cells from forming enough focal adhesions to fully polarize themselves with the axis of the gradient. As a result, sprouts become more tortuous and random in their growth, although not quite to the same degree as those seen in bulk control gels suggesting that there is still a sufficient gradient to elicit some directional response. Vascular sprouts eminating from aggregates seeded in the middle region of the gradient exhibited mixed characteristics that fell between those seen in the top and bottom regions of the gradient hydrogels. Based on these findings, gradient properties could be tailored to achieve a specific degree of directionality during vascular sprout formation.

To the best of our knowledge, this is the first instance investigating the effects of gradients of matrix mechanical properties and immobilized ECM molecules on directed 3D cellular behavior and neovascularization in proteolytically degradable PEGDA hydrogels. While the use of simultaneous gradients to direct 3D vascular sprout formation may potentially lead to more rapid and enhanced neovascularization of synthetic tissue engineered constructs, the specific role of each gradient in this process needs to be further evaluated. In this study gradients of crosslink density were inherently linked to gradients of MMP-sensitivity and degradation since the MMP-sensitive domains were crosslinked within the gel in order to enable 3D cell invasion into the matrix. Gradients of crosslink density (and stiffness) were also linked to gradients of YRGDS since regions exposed to a higher degree of crosslinking also resulted in a higher incorporation of pendant YRGDS moieties. Future experiments will attempt to isolate the effects of individual gradients of elastic modulus, immobilized concentrations of YRGDS, or MMP-sensitivity on the observed directional response by the manipulation of the composition of specific precursor components in the feed stream to the perfusion system. Furthermore, to gain a better understanding of the distance that cells are able to sense a particular gradient, additional control groups with uniform properties similar to those in different regions of the gradient hydrogels will be investigated in future studies of scaffolds presenting individual gradients. Finally, future efforts will address the effects of variations in the magnitude of the gradients on directed neovascularization.

### Conclusion

In this study, we quantified vascular sprout formation in PEGDA scaffolds that presented simultaneous gradients of elastic modulus, immobilized concentrations of YRGDS and MMP-sensitivity using an *in vitro* neovascularization assay. In scaffolds containing these gradients, bi-directional sprout invasion was observed with vascular sprouts oriented both up and down the gradient. Vascular sprout directionality was more prominent in gradient regions of increased stiffness, as well as increased presentation of crosslinked MMP-sensitive domains, and immobilized YRGDS concentration. The presented study offers insight in designing synthetic scaffolds that can be used to promote directed and guided neovascularization within engineered tissues.
